# From Yasashii Nihongo in non-disaster times towards a plurilingual language education approach: an outlook from the perspective of “reasonable accommodation”

**DOI:** 10.12688/f1000research.36372.2

**Published:** 2021-03-19

**Authors:** Hideaki Ito, Alexander Tokarev

**Affiliations:** 1 Faculty of Humanities and Social Sciences, University of Tsukuba, Tsukuba, Ibaraki, 3058577, Japan; 2 Independent Researcher, Cologne, Germany

**Keywords:** Yasashii Nihongo, plurilingualism, multicultural coexistence, reasonable accommodation, disability

## Abstract

In order to address labor shortages, starting April 2019 the Japanese government introduced two new visa categories, and it can be expected that the growing number of foreign residents living and working in Japan will be increasing further in the foreseeable future. Within this context, the notion of Yasashii Nihongo or Simplified Japanese has been gaining attention over recent years. Originally designed as a tool for transmitting information in disaster-related situations and proposed for disaster mitigation purposes, at present it is being advocated as a means of communication to be used in non-disaster situations as well. The authors argue that ultimately Yasashii Nihongo for non-disaster situations may be just a means to an end. Seen from the perspective of “reasonable accommodation”, a concept prevalent in the domain of disability studies, they assert that by de facto creating a new linguistic category making it a tacit prerequisite to communicate in “Japanese only”, Yasashii Nihongo is but a concept geared towards the language majority (speakers using Japanese as their first language) and is potentially serving no other purpose than to alleviate the psychological burden of having to speak in a language other than Japanese, thus potentially leading to a new form of discrimination towards language minorities. Offering an alternative approach for improving multicultural communication aimed at establishing a communicative space based on openness, equality, and mutual respect for each other’s cultural, linguistic and ethnic identities, the authors propose the introduction of language education based on the notion of plurilingualism, as outlined in the Common European Framework of Reference for Languages (CEFR) by the Council of Europe.

## Introduction

At present, there are a growing number of foreign residents living in Japan. There has been for a number of reasons also an increase in children who have need of formal Japanese language education (JLE), and all things to do with JLE have become an increasingly popular topic over the recent years and are covered by Japanese media outlets on a daily basis. On April 1, 2019, the Japanese Government established a new visa category for foreign personnel. It is, therefore, to be expected that the number of foreign residents in Japan will be increasing further in the foreseeable future as well. Communication barriers resulting from difficulties understanding the Japanese language, however, have been becoming noticeable already, and in this context, Simplified Japanese or Yasashii Nihongo has been steadily gaining attention.

Yasashii Nihongo denotes a variety of the Japanese language that has been simplified so that foreigners can understand it better. Originally, the notion of Yasashii Nihongo was introduced by the Sociolinguistics Department of Hirosaki University in 1999 as a response to the Great Hanshin earthquake of 1995 in Kobe, when crucial disaster-related information failed to reach people who didn’t speak English or Japanese. Seen within this context, such Yasashii Nihongo is also referred to as “Yasashii Nihongo for Disaster Mitigation”. Recently, a derivational form of Yasashii Nihongo has become prominent which is also used in daily life and is no longer reserved for disaster-related situations only. Various attempts or movements to utilize Yasashii Nihongo in non-disaster times as a tool of communication within the context of Japan’s multicultural coexistence policy ^1^ ( *tabunka kyosei*) can be observed ( Iori, 2009). However, can a construct that had been originally developed with the “primary goal of accurately transmitting information to persons affected by natural disasters” ( Sato, 2016: 256) and goes back to an adopted variety of the Japanese language specifically designed for purposes of disaster mitigation that “aims to offer safety and empowerment to victims of natural calamities” ( Sato, 2016: 272) be really considered an appropriate and efficient means of communication that could help successfully foster a truly multicultural society? Sato (2016) states that Yasashii Nihongo designed for disaster mitigation may run risk of being treated as a joke when applied to everyday life and even be a source of discrimination and potentially lead to conflict in other contexts as well; he concludes that a disaster situation after all represents a very different kind of communicative context altogether than that of a non-disaster situation. Iori (2009) explains why Yasashii Nihongo is nonetheless being increasingly used in non-disaster contexts as follows: “While we recognize that the multilingualization is an ideal to aim for, it is also a truism that de facto you need to be able to communicate in Japanese.” This prerequisite stems from an alleged “half-way resignation” towards a society where at present multilingualization may not always be deemed a viable option.

As Iori (2009) states correctly, from the standpoint of guaranteeing access to information, devising a means of communication able to provide information to a large number of people is obviously of great importance. However, creating a new linguistic category of Yasashii Nihongo to be used in non-disaster situations may not necessarily be the best option for that and might potentially even lead to discrimination against persons who are a language minority. Regarding this matter, Iori cites Tanaka (1989: 24) who touches upon the subject and illustrates how dialects are often perceived as inferior structures to standard Japanese and elaborates that this “inferiority doesn’t necessarily have to do with language in and of itself, but is rather construed as such by society which happens to see it that way.” According to Iori (2009: 129), Yasashii Nihongo has been devised to function as a variety of Japanese that is easier to understand; Iori asserts that the more it spreads, the faster Yasashii Nihongo should be able to lose its stigma of being a “second-rate language”. The present authors would like to raise the question if that is realistic, as potentially this could very well only lead to a new kind of language discrimination in the form of a mental shift within certain persons in society from criticizing someone simply because they don’t understand Japanese to criticizing someone because they are not even capable of understanding simplified Japanese. In the opinion of Tanaka (1989: 24), “the important thing isn’t to create a new or different kind of Japanese, but instead to improve on the user-friendliness of the already existing language”. This suggests that Tanaka, too, seems to believe that it shouldn’t be about creating a new linguistic category, but rather about “rethinking” the language that is being used at present. In this paper, the authors would like to reevaluate the category of Yasashii Nihongo used for non-disaster situations and discuss what qualities a successful means of communication should possess in contemporary Japan where the number of foreign residents has been steadily growing.

## Why Yasashii Nihongo for non-disaster situations isn’t receiving more traction

In the past few years, a number of guidebooks on Yasashii Nihongo have been published by various municipal authorities throughout Japan. Among other things they contain rules and formalized suggestions on how to rewrite or verbally paraphrase content, so it is easier to understand. On December 3, 2020 when we performed a Google search using the keywords “Yasashii Nihongo + iikae (verbal paraphrasing)”, we received around 257,000 hits, and about 182,000 results for “Yasashii Nihongo + kakikae (rewriting)”.

From these numbers alone we can conclude that at least prima facie there seems to be a considerable amount of information dealing with those topics. Ito *et al.* (2020) carried out a survey in K City (Ibaraki Prefecture) asking 180 Japanese residents aged 10–80 years if they knew what Yasashii was. Only 5 respondents (2.8%) said “I know what it is and I am using it consciously”, while 9 respondents (5%) replied with “I know what it is, but I am not using it.” A total of 31 persons (17.2%) answered “I have heard about it, but I couldn’t tell what it is.”, while 135 respondents (75%) replied with “I don’t know”. This suggests that more than 90% of the residents who took part in the survey did not know much about Yasashii Nihongo at all. This is the same trend observed in the survey by the Agency for Cultural Affairs, Government of Japan (2020). Furthermore, in Ito *et al.* (2020) survey, 81 respondents (45%) stated that "Japanese residents should learn the native language(s) of foreign residents more", while 145 respondents (81%) stated that "foreign residents should rather learn more Japanese, thus indicating a bias towards assimilation. Matsuo (2011) surveyed 213 Japanese residents in Osaka Prefecture and found a similar trend. Of course, the scope of the aforementioned study is rather limited so we do not attempt to make any comprehensive statements. However, first, we do wonder why Yasashii Nihongo isn’t very well known and second, why it isn’t being applied more frequently, even though there seems to be so much information available on this topic. While there may be a variety of reasons as to why Yasashii Nihongo isn’t more widespread, the authors believe that with future endeavors awareness regarding Yasashii Nihongo can be fostered further by various measures which would, however, go beyond the scope of this paper. What we would like to focus on here is why there seem to be persons who aren’t using it, even though they may be well aware of its existence.

Let us therefore first briefly become acquainted with the rules of Yasashii Nihongo as laid out by the Sociolinguistics Department of Hirosaki University (1999). On their official homepage, Yasashii Nihongo is subdivided into two distinct categories. Category I aims to support communicative contexts that occur within 72 hours after an outbreak of a natural calamity while Yasashii Nihongo under category II is to aid all types of communication processes situated in daily life even after 72 hours have passed. Below are presented the rules conceived by the Sociolinguistics Department of Hirosaki University (1999) for category I and category II Yasashii Nihongo respectively.

### Category I

**(1)** Avoid difficult words, use easy-to-understand language instead.
**(2)** Use short sentences; keep to simple sentence structures. Use spacing between words and organize linguistic elements in such a way that the reader can recognize them easily.
**(3)** Words that are frequently used in disaster outbreak situations as well as words that can be considered common knowledge in such contexts are not to be changed in any way; they are to be used as is.
**(4)** If possible, do not use Katakana words and/or loan words.
**(5)** Do not use Romanized Japanese script.
**(6)** Do not use onomatopoeia or phonomimes.
**(7)** Choose the Kanji you want to use carefully. Also, make sure not to overuse Kanji and to always add Furigana to the ones you do use.
**(8)** Use a date and time notation that can be easily understood by foreigners.
**(9)** Nominalized verbs may be difficult to understand. Please use verb structures instead.
**(10)** Avoid using ambiguous expressions.
**(11)** Do not use double negatives.
**(12)** Try to keep sentence-final structures uniform and consistent.

### Category II

**(1) ** Avoid difficult words, use easy-to-understand language instead.
**(2)** Make sure to use spacing between words.
**(3)** Use short sentences and keep sentence structures simple.
**(4)** Use traditional commas and dots for punctuation: ‘、’ and ‘°’ respectively.
**(5)** Be careful not to overuse Kanji and always add Furigana.
**(6)** Use loan words cautiously.
**(7)** Avoid using onomatopoeia, as it may be unfamiliar to foreigners and they may potentially have a hard time understanding what is meant.
**(8)** Nominalized verbs are difficult to understand. Whenever possible, use verb structures instead.
**(9)** Avoid double negatives.
**(10)** Try not to use ambiguous expressions.
**(11)** Do not use Romanized Japanese script.
**(12)** Use crucial expressions and words as is, and paraphrase them in brackets (…).
**(13)** Try to keep sentence-final structures uniform and consistent.
**(14)** Use a date and time notation that is easily understood.

Of course, those rules are by no means set in stone ^2^, but even if they are to be understood as rough guidelines for use, just how many people would actually be able to fully implement these after skimming through the rulebook and how many would be willing to start using this kind of Yasashii Nihongo? We believe that this high level of formalization may potentially contribute to the “creation of a very different type of Japanese”, as Tanaka (1989) puts it. As has been stated in chapter 1, the 72 hours between the occurrence of a natural disaster and satisfactory access to information and aid can potentially determine the difference between life and death. When considering communication that occurs in disaster situations in a multicultural society, it is crucial to establish the extent to which information can be provided and shared quickly and efficiently between language users with only limited language competence. However, if we are not aware of the information that is being cut down, the point made by Yoshikai (2020) that "even just keeping sentences short can make them dramatically easier to understand" (p. 57) can be very dangerous. In contrast, Usuyama & Okamoto (2020) point out that creating content in Yasashii Nihongo does not follow any officially binding rules and by continuing to allow local governments to create their own respective versions of Yasashii Nihongo, there is a risk of affecting the very rules for writing Japanese itself. Similar to the assimilation bias found in the data by Ito *et al.* (2020), with regard to category II, it is possible that one of the main reasons for Yasashii Nihongo among speakers using Japanese as their first language could be the wish to reduce the psychological burden of communicating in a language other than Japanese. Thus, speakers not wishing to actively engage with speakers using Japanese as a 2nd language, might not be entering an environment where they would feel uncomfortable or psychologically burdened by it in the first place.

Even when the use Yasashii Nihongo is embraced, there exists a certain potential of it coming off as patronizing, instilling an awareness that the language used on a daily basis should be changed, but also that this language should still be Japanese. This would only translate to another tacit constraint to rely on “Japanese only”, also for people using Japanese as a second language. Even in situations when it remains unclear if this would constitute an appropriate approach, Sato & Murata (2018) point out that by repeatedly presenting this as a de facto standard, the same "standard" runs risk of getting reproduced over and over again until it becomes fossilized as the only option of communication leading to a potential risk of language becoming an ideology.

Besides overly complex rules of simplification, the reason for the limited use of Yasashii Nihongo may also lie in a mode of communication that excessively focuses on the question of “how Japanese should be simplified” ^3^. Such behavior runs risk of ultimately creating a language ideology completely negating the possibility of diverse communication. In that case, we believe one should strive to look for a more diverse way of communicating in society that would certainly include, but not be limited to simplified Japanese. Even when simplified Japanese is used, but someone speaking Japanese as a second language still has difficulties understanding something, the linguistic burden would remain the same. Psychologically, it might even be greater, as the recipient of Yasashii Nihongo might feel like they failed to comprehend something they should have, as it had been specifically simplified on their behalf. Wouldn’t it be a more equal approach if both parties shared the psychological burden of communicating in a foreign language amongst themselves equally by respecting each other’s linguistic identities and trying to communicate in a more flexible manner by using a greater number of potential languages instead of limiting themselves to just one?

This is of course by no means an appeal to boycott the use of Yasashii Nihongo for non-disaster situations and shouldn’t be interpreted as such. However, it also shouldn’t be about language form in and of itself. It’s rather about a subtle difference in the degree of consideration given: if you pay attention to the person you’re talking to and you get the impression that they’re having trouble following you for some reason, you might want to try and paraphrase what you’ve just said in a different manner. Or present the same language elements written in Kanji if you think this might facilitate the understanding process. Or you might want to try and say something in the other person’s language, even if you’re not completely sure that what you’re saying is correct. Ultimately, it all comes down to fostering an environment where people can freely utilize different communication strategies in a flexible manner. In this regard, Iori *et al.* (2020) have recently added that Yasashii Nihongo is not necessarily referring to any specific expression in particular, but rather represents a “mindset” that is about mutual understanding. Yoshikai (2020) further argues that Yasashii Nihongo could be used to improve the accuracy of machine translation as a tool to promote multilingual translation. However, if the core of Yasashii Nihongo is all about a “mindset” or referring to a tool for improving accuracy in machine translation, there should be no need for creating a new language category called Yasashii Nihongo altogether. After all, such a strict distinction between Japanese used by native speakers and Japanese used for communicating with speakers using Japanese as a 2nd language is, in and of itself, potential breeding ground for discrimination.

After all, shouldn’t the direction behind the ambitions of Yasashii Nihongo be about embracing a multicultural society? If so, there should be no need to cling to the notion of having to communicate in Japanese only as appears to have been stubbornly the case up to now. The focus should rather be on the question of what steps can be taken to facilitate efficient communication in a multilingual and multicultural society.

## Reasonable accommodation

Under the Convention on the Rights of Persons with Disabilities (CRPD) ratified by Japan in 2007, “persons with disabilities include those who have long-term physical, mental, intellectual or sensory impairments which in interaction with various barriers may hinder their full and effective participation in society on an equal basis with others” ( MOFA, 2014: 6). Of course, it goes without saying that according to this definition it would be quite inappropriate to subsume foreign residents living in Japan under the same category as outlined in the CRPD. However, if we were to understand the latter part (“…interaction with various barriers may hinder their full and effective participation in society on an equal basis with others”) in a broader context and consult Ishikawa’s social model of disability ^4^ ( Ishikawa, 2008: 93) according to which “a disability constitutes a barrier that occurs in relation to society” and “a person with a disability is defined as someone who encounters such a disability”, at least partially certain similarities with regard to the various barriers foreign residents may be encountering in society come to mind - as seldom they may also be a linguistic and/or cultural minority. Seen from this perspective, the authors believe that referencing the provision of the CRPD in the context of this paper may merit a second thought. Its article introduces the notion of “reasonable accommodation” which refers to “necessary and appropriate modification and adjustments not imposing a disproportionate or undue burden, where needed in a particular case, to ensure to persons with disabilities the enjoyment or exercise on an equal basis with others of all human rights and fundamental freedoms.” ( MOFA, 2014: 7).

Persons with a visual disability and persons with a hearing disability would probably have a very different reaction when handed a piece of information written on paper and asked to read it. It is clear that a one-size-fits-all approach to providing accommodation does not work, and yet due to attempts to cut down costs, accommodation that would be specifically tailored to the needs of each and every individual with a disability is oftentimes denied. Even if an “accommodating” gesture is provided along the way, such accommodation cannot be regarded as reasonable. Denying reasonable accommodation to persons with a disability considerably limits their ability to participate in society and according to the CRPD constitutes a form of discrimination on the grounds of disability ^5^. Ishikawa (2008) elaborates on how oftentimes the notion of reasonable accommodation is misunderstood and provides the following example: if it’s hard to hear a public speaker talk clearly, because the microphone isn’t working properly or hasn’t been set up correctly, it would be obvious to assume that this would be either the fault of the lecturer or the organizer. Obviously, this issue has to be fixed and naturally, no one would even think to call this corrective measure as being considerate or especially accommodating as it is self-evident that the attendees have the right to be given access to information. For some reason the same situation is treated very differently, for example when a sign language interpretation is provided for people with a hearing disability. We are all context- and environment-dependent beings to varying degrees, but strangely the simple fact of respecting the needs of a minority is suddenly viewed as being particularly considerate or especially accommodating. It is not, and to rectify this kind of inequality in how accommodation is perceived and offered is what reasonable accommodation is all about ( Ishikawa, 2008: 94).

While the Kyushu Federation of Bar Associations and Oita Prefectural Bar Association (2017) recognize that there are differences between the Japanese language skills of foreign workers and what constitutes a disability, they believe that given past violations against obligations to consider safety, it is meaningful for a similar concept of reasonable accommodation to be developed and introduced for foreign workers. Mashiko (2018) states that while approaches in special needs education do represent a different domain, structurally there may exist certain similarities to the situation of Japanese language learners in language education: every endeavor undertaken is ultimately about finding specific ways of meeting the diverse and multifaceted needs of each individual.

The important thing when discussing disability is above all to focus on the people with a disability: to listen to what they have to say about it themselves, find out what their desires are, what they need, how they perceive it, what they think about it, what their personal experience has been. Only then can support be efficiently provided and reasonable accommodation realized. Foreign residents living in Japan may also encounter various barriers in their everyday lives. And while their struggles and concerns are very much multifaceted and should hardly be generalized, the type of support and accommodation they have been provided up to now is marked by a high level of formalization and regulation. By implicitly making it mandatory for residents whose first language is other than Japanese to use Japanese in Japan, the majority also denies the minority support and aid in their respective first language(s), thus de facto dictating what kind of accommodation the minority is entitled to receive. This may just be actively creating a further barrier hindering the participation in society of a minority. Beacco (2018) notes that “often immigrants are obliged to learn the language of the host society, that is the national and/or official language of a language majority, not always solely based on practical motives, but also oftentimes out of purely ideological reasons”. Beacco (2018: 326) states that such a standing of inequality only establishes further disadvantages to do with whether you are able to speak the language of the host society or not and should be revised from the standpoint of reasonable accommodation. To an extent, it may be possible to “overcome” a disability and indeed taking measures to compensate for a disability in order to alleviate the degree of inconvenience ( Ishikawa, 2000) may appear to be a sensible approach. However, it can also be unhealthy if one is persistently reminded by one’s environment to demonstrate that they have been indeed actively trying hard to overcome their disability or having to repeatedly present such “proof of ability”, oftentimes at the cost of their own emotional well-being. Making a conscious effort to overcome one’s disability, at least in theory, should at some point contribute to the mitigation of inconvenience, but if that action has no intrinsic value, is potentially forced and serves solely to prove to a third party that one belongs indeed to the category “overcomer”, the efforts invested in such endeavors may appear out of proportion.

Ishikawa (2000: 598) points out that such a status quo would de facto translate to the notion of a sort of “moral obligation or moral responsibility that people with a disability must assume” regardless of whether they want to or not. Communication that is carried out under the postulated framework of “Japanese-only” likewise results in a tacit claim for “proof of ability” and is liable to produce a number of disheartened individuals who might potentially start blaming themselves for their perceived linguistic shortcomings. It is easy for a society geared towards a majority to demand convenience and comfortable solutions. However, the moment the minority group concedes to the demands of the majority and adapts in a way that the majority wants it to, the moment it has to adjust to a greater extent than it feels it actually needs to - the minority group becomes subjugated to the prescriptivism of the majority. Beacco (2018: 236) states that such linguistic behaviors run risk of becoming the de facto norm, when the host society interprets such concessions as a token of gratitude and acceptance, thus reminding the minority once more and reinforcing the notion that this is exactly the way they expect them to act. According to Nagase (2002), one historically prominent fallacy in disability studies has been the rejection of a multifaceted self; rather assumptions about the individual were based on the grounds of a narrowly defined notion of normalism, oftentimes possibly invoking a feeling of shame or guilt in persons with a disability as a consequence and potentially making them believe that they might be somehow at fault for their perceived shortcomings, and that they might be a burden to society ( Nagase, 2002: 144). However, it can be observed that recently there has been a paradigm shift. “I'm not inherently wrong for being someone with a disability, society is wrong for not accepting me the way I am.” ( Nagase, 2002: 145) is a way of reframing the situation. Abe (2010: 292–293) comments on this idea and states that of course, while there may be some things people with a disability might have trouble doing and while some might argue it were the society oftentimes “disabling” these individuals, he postulates that in certain contexts everyone would be affected similarly. Abe provides the following example to illustrate his point: “When you fail to comprehend the modus operandi of a particular discourse, when you are unable to understand certain words used therein, just about everyone would have a hard time following, and participating in a communication process like that.” Facing such adversity, it might be quite difficult to get a sense of belonging or to experience oneself as being a respected member of a group. The use of expressions that are easily understood is a key element in creating a communication culture that is characterized by equality and mutual respect ( Abe, 2010: 303). This does not only hold true for the Japanese language community on Japanese as a second language, but for other sociolinguistic contexts as well. Once Japan has transformed into a genuinely multilingual society, it will become a commonplace occurrence in daily life to encounter a variety of expressions being used in languages other than Japanese. And when that happens the majority in the Japanese society, that is either speakers who are only able to communicate in Japanese exclusively or those who rely heavily on the use of English, may in turn face similar communication issues: they would then experience first-hand what it feels like to be facing a communication barrier simply because they don’t understand a certain language or languages. It goes without saying that such communication mishaps run risk of creating a major rift between all parties involved. To prevent the formation of this communication gap it is vital to create more opportunities within society for its participants to be able to learn other languages. This way, native speakers situated in the host society, as well as speakers speaking Japanese as a second language could become avid learners of foreign languages, communicating using a variety of languages, oftentimes intermingling two or more languages at a time.

## Plurilingualism-based education approaches

As has been stated in the previous sections regarding the use of Yasashii Nihongo for non-disaster situations in a multicultural and multilingual society, the authors believe the currently predominating focus on the philosophy of “Japanese-only” to be a rather suboptimal solution, especially if seen from the standpoint of reasonable accommodation. It would be more desirable to try and realize a more equal model of communication where several languages could be used in combination with each other and elements from a variety of languages be intermingled. The question remains: what measures could be taken to make this a reality? How could this concept be practically implemented? The authors believe the key may lie in the idea of plurilingualism as proposed in the Common European Framework of Reference (CEFR) published by the Council of Europe in 2001. Kawakami & Ozeki (2010: 80) offer a definition of plurilingualism in relation to its entities: individuals are plurilingual if they possess knowledge and experience of a variety of languages (=and cultures) and are able to combine and utilize both in a flexible manner depending on the communicative context (e.g. the persons they are communicating with). Accordingly, language education that is based on such a notion of plurilingualism does not aim to foster native-like language proficiency; in fact it does not even need to operate using the terms native language or mother tongue and 'native culture' or 'mother culture', respectively. Rather, it welcomes learners with different experiences from different backgrounds, and it is about cultivating learners with the competence and ability to respond to various situations at different times by engaging in communicative acts, which is to mean the ability to take action by means of communication. According to Okumura *et al.* (2016:21), the more languages you know, the easier it becomes to be in control of the communication even when confronted with an unfamiliar situation.

Plurilingualism is empowering. It enables people to live and take action independently as diverse members of society. This is why in Japan, too, it might be a good idea to avoid making it a tacit prerequisite to communicate in Japanese only or limit communication to one particular language in general. Instead, adopting an attitude of openness towards the language(s) of the interlocutor may have a much more desirable effect. For instance, when exchanging greetings and pleasantries, but also in other daily life contexts, one could try and mix Japanese with the first language of the person or persons one is talking to, so as all parties involved would share the weight of the linguistic and psychological burden of communicating in a foreign language equally, thus fully mobilizing each other’s individual language potentials. Ultimately, such a more diverse type of communication would be an essential contribution to making the sociolinguistic reality richer. For this, we believe it is necessary to establish a new culture of language education based on the notion of plurilingualism. Candelier (2018) proposes the notion of “pluralistic approaches” (approches plurielles), describing a didactic system based on a multilingual framework of education. He identifies four distinct pluralistic approaches and subdivides these into the following groups (ibid). One includes the so-called ‘awakening to languages’ method, when at least a number of the interventions undertaken in educational settings touch upon languages that are not necessarily formally taught at school. Within the model of ‘inter-comprehension between related languages’, several languages of the same linguistic family are studied in parallel. The ‘integrated teaching’ approach is geared towards empowering learners to develop links between elements from a certain number of languages that they had acquired previously in formal educational settings. Last but not least, ‘intercultural education’, possibly one of the better known models of the four, strives to offer insights from the perspective of multiple cultures simultaneously. In this paper, the authors propose the promotion of an ‘awakening to languages’, a pluralistic education approach that they believe is much needed within contemporary Japanese society to foster a new culture of communication based on the possibility of utilizing several languages simultaneously. Oyama (2016) states that even if the working language were Japanese, within a framework of ‘awakening to languages’ at school, educational activities aimed at discovering similarities between multiple languages would benefit all parties involved, as long as they are carried out free of any position of perceived sociolinguistic superiority. If languages that are usually not taught in primary or secondary education, such as Tagalog or Portuguese, were given a chance to be explored and experienced by carrying out activities at school based on the ‘awakening to languages’ approach, not only would the children from language minorities discover new confidence in their own respective languages and cultures, but this would also lead to a culture of more openness and acceptance towards diversity in general, helping to deconstruct possible preconceived notions on the part of the language majority ( Oyama, 2016: 18). While activities based on the ‘awakening to languages’ approach are rarely implemented in Japan, Oyama (2016) portrays its potential use cases and implications in his work in more detail. Traditionally, Japanese and English have been the two languages given utmost priority within contemporary Japanese society ( Kimura (2016) states that over-reliance on English is more of a problem). In this paper, the authors argue that in the near future young professionals who will have had the chance to experience, learn about, but also learn from cultural and linguistic minorities will become an indispensable source of human potential, regardless of their nationality or the fact that Japanese has been their first language or not. To foster such positive awareness of diversity, the authors believe it is essential to take full advantage of the learning opportunities found within formal school education and community-based regional Japanese language classes and thus create further opportunities for young people to become more engaged in learning about and getting acquainted with various foreign languages. As has been stated previously, language education based on the notion of plurilingualism doesn’t necessarily aim to produce language speakers who - from a linguistic point of view- would be capable of dealing just with any situation imaginable as proficiently as a native speaker might. Rather, it is a form of language education that encourages openness and diversity and teaches to adapt to an increasingly diverse society in order to live together with people from various personal backgrounds in a more harmonious way. For those even hesitant to try Yasashii Nihongo, the idea of plurilingual education may seem like an even larger hurdle. The point of this paper, however, is by no means that the use of Yasashii Nihongo should be discouraged. In this paper, we rather argue that Yasashii Nihongo should not be treated as a separate entity of the Japanese language, but rather function as a form of “Yasashii Language”, promoting various kinds of interpersonal interactions with the primary aim of adjusting the language itself to accommodate the other person – no matter what language is used. By participating in everyday activities drawing from a diverse landscape of various languages while not being limited exclusively to Japanese or another single language, a new mode of learning can emerge that would have the potential to overcome discrimination and prejudice for all parties involved, regardless of one’s first language being Japanese or another language. Such a ‘community of practice’( Lave & Wenger, 1991) would ultimately foster a Japanese society more open to linguistic and cultural diversity.

Seen from the context of natural calamities, it might be necessary to establish functional methods of guaranteeing access to information as a means of disaster mitigation, and Yasashii Nihongo for disaster situations would be of course a fitting solution for that. At least with regard to contexts occurring in daily life, however, it would be crucial for the future Japanese society if communication were to be carried out in such a way that all parties involved would share the weight of the linguistic and psychological burden of communicating in a foreign language equally while respecting the diversity of each other’s languages and cultures by having the chance to learn and get acquainted with each other’s respective languages.

## Conclusion

The number of foreign residents living and working in Japan has been growing steadily. In this paper, we have devoted our attention to the communication between the language majority (speakers using Japanese as their first language) and language minority (foreign residents with a first language other than Japanese) in contemporary Japanese society. We have drawn attention to the potential perils that go hand in hand with making “Japanese only” a tacit prerequisite for communication in a multicultural society, and we illustrated the necessity of rectifying the underlying inequality behind differing degrees of accommodating processes present in daily life, as both the language majority and language minority being equal constituent members of society should have the right to be building it on an equal footing side by side. As a promising approach to creating a truly multicultural and multilingual environment, we have proposed the introduction of a model of language education based on the notion of plurilingualism while putting a special emphasis on that a certain degree of flexibility is always desirable when engaging in such communicative contexts. With regard to current developments within the status quo of Japanese society, awareness must be raised to establish a more equal form of intercultural communication lest the majority takes on an overly dominating role. A first step to prevent such development may lie in the language majority becoming self-conscious about being the majority and making an active endeavor to act mindfully of the voices belonging to those in the language minority. The authors hope that this paper has been insightful in raising awareness to the positive potential of a new plurilingualism-based model of multicultural and multilingual communication in contemporary Japan.

## Data availability

No data are associated with this work.

## Notes

^1^ The term *tabunka kyosei* (literally, 'many cultures living together') became prominent in 2006, when the Ministry of Internal Affairs and Communication (MIC) issued a plan for the “promotion of multicultural coexistence” in local communities. Its aim is among other things to create an environment where “persons of various nationalities, races and ethnic identities live together as constituent members of local communities while recognizing each other’s cultural differences and attempting to establish equal relationships.” ( MIC, 2006: 1).

^2^ According to Usami (2014), there exists a certain creative freedom as to following the proposed guidelines and the sequence that its individual entries are listed.

^3^ The act of simplifying Japanese to express an idea in simpler terms is not at all problematic in and on itself. Tanaka *et al.* (2013) presents an interesting showcase of Yasashii Nihongo used within the domain of news reporting and writing while Uchinami (2018) addresses the positive potentials Yasashii Nihongo can unfold when applied to empower persons with a cognitive disability.

^4^ The hitherto predominant ‘medical model’ does not relate to society as such. Instead it’s structured around the notion of whether a functional disability is present in an individual and to what extent it can be rectified using medical countermeasures.

^5^ While individuals should be careful to avoid being discriminatory as well, the scope of its anti-discrimination statute is limited to governmental institutions, administrative bodies and public entities only.
