# Supplementary material for: From Yasashii Nihongo in non-disaster times towards a plurilingual language education approach: an outlook from the perspective of “reasonable accommodation”
Source: F1000Res. 2021 Mar 19;10:52. Originally published 2021 Jan 28. [Version 2] doi: 10.12688/f1000research.36372.2 (PMC8063522; doi:10.12688/f1000research.36372.2)
Supplement: Peer review history

# Peer review: Reviewer response for version 2

**DOI:** 10.5256/f1000research.55367.r81739

**Published:** 2021-04-20

**Authors:** Goro Christoph Kimura

**Affiliations:** 1 Sophia University, Tokyo, Japan

Criticizing the attempts to promote Yasahii Nihongo (Simplified Japanese) as a distinct category of Japanese within a monolingual framework, this paper proposes to integrate it as part of plurilingualism, according to which communication will not be confined to just one language. The approach of reasonable accommodation provides an additional theoretical background to this argument. This is a welcome contribution to the discussion on linguistic aspects of intercultural communication in Japan.

Is the topic of the opinion article discussed accurately in the context of the current literature?

Partly

Are arguments sufficiently supported by evidence from the published literature?

Partly

Are all factual statements correct and adequately supported by citations?

Partly

Are the conclusions drawn balanced and justified on the basis of the presented arguments?

Yes

Reviewer Expertise:

sociolinguistics, interlingual communication

I confirm that I have read this submission and believe that I have an appropriate level of expertise to confirm that it is of an acceptable scientific standard.

# Peer review: Reviewer response for version 2

**DOI:** 10.5256/f1000research.55367.r81738

**Published:** 2021-03-23

**Authors:** Anthony J. Liddicoat

**Affiliations:** 1 Department of Applied Linguistics, University of Warwick, Coventry, UK

The writers have responded suitably to the previous review by developing a more critical discussion of the Yasashii Nihongo provisions of Hirosaki University. It has not contextualised the phenomenon of Yasashii Nihongo in other language planning work for simplified registers.

Is the topic of the opinion article discussed accurately in the context of the current literature?

Yes

Are arguments sufficiently supported by evidence from the published literature?

Yes

Are all factual statements correct and adequately supported by citations?

Yes

Are the conclusions drawn balanced and justified on the basis of the presented arguments?

Yes

Reviewer Expertise:

language planning and policy, language education

I confirm that I have read this submission and believe that I have an appropriate level of expertise to confirm that it is of an acceptable scientific standard.

# Peer review: Reviewer response for version 1

**DOI:** 10.5256/f1000research.39434.r79158

**Published:** 2021-02-19

**Authors:** Anthony J. Liddicoat

**Affiliations:** 1 Department of Applied Linguistics, University of Warwick, Coventry, UK

This article presents an opinion about the role of Yasashii Nihongo in contemporary Japan and argues that the dissemination of Yasashii Nihongo is potentially problematic because it is more predicated on the needs of Japanese speakers than those of immigrants/foreigners. It then advocates for a plurilingual approach in Japan based on ideas drawn from the CEFR and éveil aux langues. It links such approaches to the notion of reasonable adjustment and launches an interesting idea about what such a concept might mean in the context of language.

 Overall, this paper makes some interesting points. I would have liked to see a more critical discussion of the Hirosaki University’s descriptions for category one, which are generic and very open to interpretation. And takes an interesting position in relation to what constitutes ‘easy’ in relation to Japanese. The paper also focuses on Yasashii Nihongo as if it were a unique phenomenon rather part of a larger language movement (Plain English, français pour tous, Vereinfachtes Deutsch, Español Simplificado Internacional, etc.), with similar motivation of simplifying communication while preserving monolingual practice.

Is the topic of the opinion article discussed accurately in the context of the current literature?

Yes

Are arguments sufficiently supported by evidence from the published literature?

Yes

Are all factual statements correct and adequately supported by citations?

Yes

Are the conclusions drawn balanced and justified on the basis of the presented arguments?

Yes

Reviewer Expertise:

language policy, language education

I confirm that I have read this submission and believe that I have an appropriate level of expertise to confirm that it is of an acceptable scientific standard, however I have significant reservations, as outlined above.

# Response

**Published:** 2021-03-10

**Authors:** Hideaki Ito

**Affiliations:** University of Tsukuba, Japan

Thank you for your valuable comments.

 1) Regarding the discussion in category 1 and language movements:

 Thank you for your comments from a broader perspective beyond the domestic movement in Japan. In Section 2, we have added a note on Category 1, referring to the global movement and citing Yoshikai (2020) and Usuyama & Okamoto (2020).

# Peer review: Reviewer response for version 1

**DOI:** 10.5256/f1000research.39434.r78440

**Published:** 2021-02-16

**Authors:** Goro Christoph Kimura

**Affiliations:** 1 Sophia University, Tokyo, Japan

In contemporary Japan, where the number of foreign residents has been steadily growing, Simplified Japanese or Yasashii Nihongo has been steadily gaining attention. The authors argue that Yasashii Nihongo may be useful in immediate disaster situations, but creating a new linguistic category of Yasashii Nihongo to be used in every-day situations may not necessarily be the best option and might potentially even lead to discrimination against persons who are a language minority, forcing them to use Japanese only. They point to the possibility that Yasashii Nihongo can be used as a pretext by some members of the linguistic majority in Japan not to use any foreign language and discriminate those who cannot “even” understand simplified Japanese. Thus, the psychological burden of having to communicate not in the first language is laid on the side of the migrants. According to the authors, the notion of “reasonable accommodation” which calls for attention to individual needs of those suffering from disability caused by the society, can be utilized also to the communication between Japanese native speakers and foreign residents. Instead of trying to create an entirely novel category of Japanese with fixed rules of simplification, the authors propose a plurilingual approach that allows using various languages flexibly. They argue that adopting an attitude of openness towards the language(s) of the interlocutor may have a much more desirable effect than adhering to the use of one language. To enhance such a plurilingual attitude, the authors propose the promotion of an ‘awakening to languages’ that seeks to create positive awareness of linguistic diversity.

 Research on Yasashii Nihongo is largely dominated by uncritical supporters of this approach. So this critical study is a welcome and indeed needed contribution to the field of research. The alternative proposed in this paper, plurilingual communication based on language awareness, shows a desirable way out of the monolingualism and normalism to which an emphasis on Yasashii Nihongo as a codified form of simplified Japanese could lead. The arguments comparing the situation of speakers of Japanese as a second language with persons with disabilities are convincing and can contribute to further elaborate the emergent discussion on “disability-oriented language rights” (Kimura, 2019 ^1^).

 Providing such inspiring insights, it is regrettable that the authors seem not to have followed the latest developments in the study on Yasashii Nihongo. First, they heavily rely on early forms of Yasashii Nihongo for disaster situations, which was inclined to set fixed rules, to criticize Yasashii Nihongo in general. Recent developments, however, tend to suggest improvements in various situations, rather than proclaiming fixed rules (see for example Iori (ed.) 2020). Second, it is doubtful whether a philosophy of “Japanese-only” is so prevalent in the discourse of Yasashii Nihongo. Often, information in Yasashii Nihongo is given as one option among various languages, as prominently exemplified by https://covid19-tagengo.com/. Iori (ed)(2020) mentions Yasashii Nihongo as an interlanguage for translation (p.56). Yoshikai (2020) even more directly situates Yasashii Nihongo as a tool to promote multilingualism.

 Some assertions are stated without discussing the plausibility. For example, the following sentence lacks a source supporting this argument: “Resorting to Yasashii Nihongo simply to ease one’s psychological burden of having to speak in a language other than Japanese may just have been the reason why it hasn’t been able to find more exposure.” The question “why there seem to be persons who aren’t using it, even though they may be well aware of its existence” is not pursued in this article, so still waits for an answer. The authors also question if it is realistic that Yasashii Nihongo turns into an accepted register in the use of Japanese. But they seem to be too optimistic with regard to the acceptance of plurilingualism. Why should the majority reluctant even to accommodate their Japanese language to the need of a speaker of Japanese as second language show more willingness to flexibly interact in the sense of plurilingualism? If raising awareness about language diversity could change the attitude, it could also be expected that awareness raising on Yasashii Nihongo as a strategy of interlingual communication would lead to its general acceptance. To me, enhancing plurilingualism as a general attitude in Japanese society seems even less realistic than hoping that Yasashii Nihongo will be accepted as a variant on its own right. The authors do not mention English as a relevant factor, but the over-reliance to and over-estimation of English in the Japanese society seems to impede Yasashii Nihongo as well as plurilingualism.

 The survey by the authors about the familiarity with Yasashii Nihongo could be complemented by a partly similar survey published by the Agency for Cultural Affairs, showing that about 30% of the respondents answered to know Yasashii Niihongo ( https://www.bunka.go.jp/koho_hodo_oshirase/hodohappyo/pdf/92531901_01.pdf).

 I found only one typo: (Ishizkawa 2008: 93) -> (Ishikawa 2008: 93)

 References:

- Iori, Isao (2020) *Yasashii Nihongo Hyogen Jiten*, Maruzen.
- Kimura, Goro Christoph (2019) Language rights, in: Patrick Heinrich and Yumiko Ohara (eds.): *Routledge Handbook of Japanese Sociolinguistics*, London & New York: Routledge, 389-403.
- Yoshikai Akira (2020) *Nyumon Yasashii Nihongo*, Ask Publishing.

Is the topic of the opinion article discussed accurately in the context of the current literature?

Partly

Are arguments sufficiently supported by evidence from the published literature?

Partly

Are all factual statements correct and adequately supported by citations?

Partly

Are the conclusions drawn balanced and justified on the basis of the presented arguments?

Yes

Reviewer Expertise:

sociolinguistics, interlingual communication

I confirm that I have read this submission and believe that I have an appropriate level of expertise to confirm that it is of an acceptable scientific standard, however I have significant reservations, as outlined above.

## References

[1] : Language rights, in: Patrick Heinrich and Yumiko Ohara (eds.): Routledge Handbook of Japanese Sociolinguistics. *Routledge*.2019;389-403 Reference source

# Response

**Published:** 2021-03-10

**Authors:** Hideaki Ito

**Affiliations:** University of Tsukuba, Japan

Thank you very much for your very constructive and useful comments.

 1) Regarding the reference to the latest research:

 Thank you very much for your valuable comments on the references to the latest research. In Section 2, we mentioned Iori et al. (2020) and Yoshikai (2020), and stated that there is no need to create a new language category based on the notions of "mindset" and "improving the accuracy of machine translation".

 2) Regarding the leap in argument and Optimism about Plurilingualism Education:

 Thank you for your insightful comments. As you pointed out, there were a lot of definitive expressions, so we changed the overall description to point out the possibility. As for the point that it may be a practical hurdle to promote plurilingualism rather than to accept Yasashii Nihongo, we have added an argument for using language as “Yasashii language" in Section 4, rather than having a language category called Yasashii Nihongo. Also, we recognize that the over-reliance on English is an important point. We have touched on this point in the text, citing Kimura (2016).

 3) About data from the Agency for Cultural Affairs, Government of Japan (2020):

 Thank you very much for your valuable data. In addition to this data, we have cited data from Ito et al. (2020) as reinforcement.

 4) Regarding typos:

 Thank you for pointing this out. We have corrected it.
